# Comparable Initial Engagement of Intracellular Signaling Pathways by Parathyroid Hormone Receptor Ligands Teriparatide, Abaloparatide, and Long‐Acting PTH

**DOI:** 10.1002/jbm4.10441

**Published:** 2021-05-06

**Authors:** Tadatoshi Sato, Shiv Verma, Ashok Khatri, Thomas Dean, Olga Goransson, Thomas J Gardella, Marc N Wein

**Affiliations:** ^1^ Endocrine Unit, Department of Medicine Massachusetts General Hospital, Harvard Medical School Boston MA USA; ^2^ Department of Experimental Medical Science Lund University, Diabetes, Metabolism and Endocrinology Lund Sweden

**Keywords:** MOLECULAR PATHWAYS, REMODELING, BONE MODELING AND REMODELING, PTH/Vit D/FGF23, CELL/TISSUE SIGNALING, ENDOCRINE PATHWAYS, OSTEOCYTES, CELLS OF BONE, ANABOLICS, THERAPEUTICS, HORMONE REPLACEMENT/RECEPTOR MODULATORS, THERAPEUTICS

## Abstract

Multiple analogs of parathyroid hormone, all of which bind to the PTH/PTHrP receptor PTH1R, are used for patients with osteoporosis and hypoparathyroidism. Although ligands such as abaloparatide, teriparatide (hPTH 1‐34 [TPTD]), and long‐acting PTH (LA‐PTH) show distinct biologic effects with respect to skeletal and mineral metabolism endpoints, the mechanistic basis for these clinically‐important differences remains incompletely understood. Previous work has revealed that differential signaling kinetics and receptor conformation engagement between different PTH1R peptide ligands. However, whether such acute membrane proximal differences translate into differences in downstream signaling output remains to be determined. Here, we directly compared short‐term effects of hPTH (1‐34), abaloparatide, and LA‐PTH in multiple cell‐based PTH1R signaling assays. At the time points and ligand concentrations utilized, no significant differences were observed between these three ligands at the level of receptor internalization, β‐arrestin recruitment, intracellular calcium stimulation, and cAMP generation. However, abaloparatide showed significantly quicker PTH1R recycling in washout studies. Downstream of PTH1R‐stimulated cAMP generation, protein kinase A regulates gene expression via effects on salt inducible kinases (SIKs) and their substrates. Consistent with no differences between these ligands on cAMP generation, we observed that hPTH (1‐34), abaloparatide, and LA‐PTH showed comparable effects on SIK2 phosphorylation, SIK substrate dephosphorylation, and downstream gene expression changes. Taken together, these results indicate that these PTH1R peptide agonists engage downstream intracellular signaling pathways to a comparable degree. It is possible that differences observed in vivo in preclinical and clinical models may be related to pharmacokinetic factors. It is also possible that our current in vitro systems are insufficient to perfectly match the complexities of PTH1R signaling in bona fide target cells in bone in vivo. © 2020 American Society for Bone and Mineral Research © 2020 The Authors. *JBMR Plus* published by Wiley Periodicals LLC. on behalf of American Society for Bone and Mineral Research.

## 1 Introduction

The parathyroid hormone type 1 receptor (PTH1R) is a validated osteoporosis drug target. This receptor has two distinct physiologic ligands, PTH and parathyroid hormone‐like related protein (PTHrP). Teriparatide (hPTH 1‐34, TPTD), or PTH amino acids 1‐34, has been used to treat osteoporosis since 2002.^(^ ^1^ ^)^ More recently, abaloparatide, a synthetic analog of PTHrP, has also been approved for osteoporosis treatment. When compared directly to hPTH (1‐34), abaloparatide treatment led to greater gains in bone mineral density (BMD) and improved fracture prevention efficacy.^(^ ^2^ ^)^ Abaloparatide appears to stimulate bone resorption less potently than hPTH (1‐34), which may explain some of these beneficial effects.

Despite considerable progress, the mechanistic basis for these clinically‐important differences between hPTH (1‐34) and abaloparatide remains incompletely understood. One possibility is that the structurally‐distinct ligands induce different signaling responses due to altered modes of interaction with the PTH1R (biased agonism). Two high‐affinity PTH1R conformations, R^0^ and RG, exist.^(^ ^3^, ^4^ ^)^ These distinct receptor conformations have been associated with differential kinetic signaling profiles. Ligands (such as hPTH (1‐34) and long‐acting PTH (LA‐PTH)) that preferentially associate with the R^0^ receptor conformation tend to signal for prolonged periods, potentially from endosomes, whereas ligands (such as abaloparatide) that preferentially activate RG receptors signal more transiently, and likely from the cell surface.^(^ ^5^, ^6^ ^)^ Such quantitative differences in signaling kinetics are generally revealed by performing ligand washout assays in which the duration of the response is monitored over time after initial binding, which thus reflects the residence time of the ligand‐receptor complex. Endosomal signaling is a mechanism by which ligands activate unique signaling outputs otherwise not possible from the plasma membrane.^(^ ^7^, ^8^ ^)^ How differences between abaloparatide and hPTH (1‐34) at the level of receptor conformation engagement might translate into qualitatively distinct biologic outputs in bone cells remains unknown. Moreover, although effects observed in vivo support altered modes of action,^(^ ^9^, ^10^, ^11^ ^)^ the majority of previous studies examining these conformation‐dependent effects on signaling have utilized heterologous systems with overexpressed receptors. Therefore, it is important to interrogate signaling events downstream of the PTH1R in bone cells expressing endogenous receptors.

Osteocytes, cells of the osteoblast lineage buried within bone matrix, are the most abundant, and least understood, cell type in bone.^(^ ^12^ ^)^ Osteocytes produce paracrine factors that regulate osteoblast and osteoclast function. Two such paracrine factors are RANKL (also known as tumor necrosis factor ligand superfamily member 11 [TNFSF11]), which drives osteoclastogenesis, and sclerostin (encoded by the gene SOST), which inhibits osteoblast activity.^(^ ^13^ ^)^ Agents that inhibit RANKL (denosumab),^(^ ^14^ ^)^ or sclerostin (romosozumab),^(^ ^15^, ^16^ ^)^ are in use for the treatment of osteoporosis. PTH increases bone formation in part by reducing SOST expression by osteocytes^(^ ^17^, ^18^ ^)^; but PTH also increases RANKL expression, which can lead to excessive bone resorption.^(^ ^19^, ^20^, ^21^, ^22^ ^)^ In theory, a PTH1R ligand that differentially modulates expression of RANKL and SOST in osteocytes so as to favor net bone formation could be a highly effective bone anabolic agent.

Although it is well‐established that PTH‐mediated increases in bone formation require actions in osteocytes,^(^ ^19^, ^23^ ^)^ the signaling events downstream of the PTH1R in osteocytes have remained elusive. We recently described a new signaling pathway that relays information from the PTH1R to the nucleus in osteocytes.^(^ ^24^ ^)^ A crucial step in this cascade involves cyclic AMP (cAMP)‐mediated inhibition of salt inducible kinase 2 (SIK2). Importantly, PTH1R‐induced SIK2 phosphorylation/inactivation occurs rapidly (within 1 min of receptor engagement by hPTH (1‐34)) and then diminishes.^(^ ^24^ ^)^ Novel small molecule SIK inhibitors, such as YKL‐05‐099,^(^ ^25^ ^)^ mimic PTH action in cultured osteocytes and in vivo. By inhibiting cellular SIK2 function, PTH treatment of osteocytes leads to reductions in phosphorylation of known SIK substrates (class IIa histone deacetylases [HDACs] and cAMP response element‐binding protein regulated transcription coactivator 2 [CRTC2]) and their subsequent nuclear translocation. In the nucleus, class IIa HDACs and CRTC proteins selectively regulate distinct sets of physiologically‐important PTH target genes, including SOST (class IIa HDACs) and RANKL (CRTC2). Moreover, engagement of the PTH/SIK signaling axis in osteocytes leads to coordinated transcriptional regulation of a core group of at least 142 genes, as identified by RNA‐seq.^(^ ^24^ ^)^ Therefore, SIK inhibition is a major PTH1R signaling mechanism in target cells in bone.^(^ ^26^, ^27^, ^28^, ^29^ ^)^ However, whether differences between hPTH (1‐34), LA‐PTH, and abaloparatide exist at the level of SIK inhibition, changes in SIK substrate phosphorylation, and downstream regulation of gene expression remains completely unknown.

To test the hypothesis that abaloparatide induces distinct signaling output than hPTH (1‐34) and LA‐PTH, multiple complementary approaches were pursued. First, we used HEK293 cells expressing recombinant human PTH1R, a well‐characterized heterologous expression system. In HEK293‐hPTH1R cells, we assessed the ability of PTH analogs to induce intracellular calcium flux, internalization of PTH1R, and recruitment of β‐arrestin to PTH1R complexes on the cell surface. In parallel to our studies in PTH1R‐overexpressing HEK293 cells, we also assessed downstream signaling events induced by engagement of endogenously‐expressed PTH1R in murine Ocy454 cells, a well‐characterized conditionally‐immortalized osteocyte‐like cell line.^(^ ^30^, ^31^ ^)^ In Ocy454 cells, we compared the ability of different PTH ligands to stimulate cAMP generation and to induce the phosphorylation of protein kinase A (PKA) substrates, regulate the phosphorylation of SIK2, HDAC4/5, and CRTC2. Further downstream, we also assessed the ability of different PTH ligands to regulate well‐characterized PTH target genes using RT‐qPCR and Nanostring digital gene expression profiling (Nanostring, Seattle, WA, USA).

## 2 Materials and Methods

### 2.1 Materials/peptide synthesis

Abaloparatide was obtained from Lonza Pharmaceuticals (Basel, Switzerland). Other peptides were synthesized by the Peptide Synthesis Core facility of the Massachusetts General Hospital using conventional solid‐phase chemistry with Fmoc‐protected amino acid precursors. Peptides were purified on a prep‐C18 column using reverse phase (RP)‐HPLC (solvent A: 0.1% Trifluoroacetic acid (TFA) in water, solvent B: 0.1% TFA in acetonitrile). Purity was established to be greater than 95% by analytical RP‐HPLC, and MALDI–time of flight (TOF) mass spectrometry confirmed peptide identity and authenticity. An N‐truncated LA‐PTH analog, PTH (7‐36) (LNleHQLdWKWIQDARRRAWLHKLIAEIHTAEI.NH2) was used as a negative control. Abaloparatide was 86% peptide content. The other peptide content was estimated to be 75%, based on direct quantification of similar representative peptides by acid hydrolysis and amino acid analysis. Peptide content was accounted for in preparing working stock solutions of each peptide for all cell‐based assays.

### 2.2 Intracellular cAMP

Confluent Ocy454 cells in 96‐well plates were treated with a abaloparatide or a PTH analog at varying concentrations in a buffer of Hanks balanced salts solution (HBSS) containing 0.1% BSA and 3‐isobutyl‐1‐methylxanthine (IBMX) (2mM) for 30 min at room temperature. The buffer was then removed and the cells were lysed by placing the plate on a bed of powdered dry ice and adding 50 μL of 50mM HCl. After freezing and thawing, the lysates were diluted in water and then assessed for cAMP content by radioimmunoassay.

### 2.3 Intracellular calcium (iCa ^++^)

Signaling via the iCa^++^ pathway was assessed in stably transfected HEK‐HEK293/hPTH1R/glosensor (GP‐2.3) cells using the calcium‐sensitive fluorophore Fura2‐AM (Invitrogen, Life Tech. Grand Island, NY, USA).^(^ ^32^ ^)^ Confluent cells in a black, 96‐well plate were preloaded with Fura2‐AM (5μM) for 45 min and then unloaded in buffer for 30 min. The plate was then processed using a Perkin Elmer Envision plate reader (Perkin Elmer, Waltham, MA, USA) to monitor fluorescence emission at a wavelength (λ_em_) of 515 nm, upon sequential excitation at wavelengths (λ_ex_) of 340 nm and 380 nm. Data were recorded at 2‐s intervals for 10 s prior to, and for 150 s after ligand addition. The data at each time point were calculated as the ratio of the fluorescence signal obtained with excitation at 340 nm to that obtained with excitation at 380 nm.

### 2.4 Internalization

Internalization of ligand‐PTH1R complexes was assessed by fluorescent microscopy in stably transfected HEK293/ratPTH1R/glosensor (GR‐35) cells. The cells were cultured on glass coverslips in 24‐well plates to ~75% of confluency, and then treated with a tetramethylrhodamine (TMR)‐labeled PTH analog (100nM, TMR attached to Lys13) in HBSS containing 0.1% BSA (HB buffer) for 15 min at 21°C. The cells were then rinsed thrice with HB buffer, fixed with 4% formalin for 5 min, mounted with vector‐shield containing 4,6‐diamidino‐2‐phenylindole (DAPI) on a glass microscope slide, and viewed and digitally imaged using a Nikon epifluorescent microscope (Nikon, Tokyo, Japan) equipped with a charge‐coupled device (CCD) camera configured with SPOT imaging software (SPOT Imaging, Sterling Heights, MI, USA).

### 2.5 Measurement of internalization using GFP^pHs^ fluorescence

Receptor internalization was assessed in the HEK293 cell line stably transfected with human PTH1R‐pHluorin2‐GFP (GPG‐10 cells).^(^ ^33^, ^34^ ^)^ Confluent monolayers of cells in black‐walled, 96‐well plates were incubated in HBSS with BSA (0.1% wt/vol) and HEPES buffer (pH 7.4, 10mM). Peptides were added and wells were analyzed by recording fluorescence readouts with excitation at 485 or 405 nm and emission at 535 nm. Data were analyzed as a ratio of fluorescence intensity following excitation at 485/405 nm over the course of 90 min, and the area under the curve (AUC) values for each time course were plotted as a function of ligand concentration.

### 2.6 Live cell PTH1R recycling

Protocols were previously described.^(^ ^35^ ^)^ HEK293 cells stably expressing the pH‐sensitive GFP super‐ecliptic pHluorin (SEP)‐PTH1R were plated on poly‐D‐lysine‐coated coverslips. For live imaging, cells were maintained in HEPES buffer. Confocal time lapse imaging was performed using a Nikon A1 confocal microscope with ×60, 1.4 numeric aperture objective at 37°C. Images were acquired every 1 min. Following a 5‐min baseline acquisition, 100nM PTH or abaloparatide was added and then washed out after 5 min.

### 2.7 Recruitment of β‐arrestin

Recruitment of β*‐*arrestin was assessed in HEK293 cells stably transfected to express β*‐*arrestin2‐YFP^(^ ^36^ ^)^ and glosensor (GBR‐24 cells)^(^ ^34^ ^)^ and transiently transfected to express the hPTH1R or mDsRed‐hPTH1R in which the monomeric *Discosoma sp*. red fluorescent protein is inserted in the E2 region of the receptor's extracellular domain. Cells were treated with each PTH1R ligand and imaged by fluorescent microscopy as described above (see 2.4 Internalization). The quantification of colocalizations between β‐arrestin2YFP and TMR‐ligands were performed by ImageJ software (NIH, Bethesda, MD, USA; https://imagej.nih.gov/ij/) using Just Another Colocalization Plugin (JACoP) and indicated as cytofluorograms.

### 2.8 Immunoblotting

For immunoblotting, cells were prepared as described.^(^ ^37^ ^)^ Whole cell lysates were prepared using TNT (Tris‐NaCl‐Tween buffer, 20mM Tris‐HCl pH 8, 200mM NaCl, 0.5% Triton X‐100 containing protease inhibitor [PI], 1mM NaF, 1mM dithiothreitol [DTT], 1mM vanadate). Adherent cells were washed with ice cold PBS, then scraped into TNT buffer on ice. Material was then transferred into Eppendorf tubes kept on ice, vortexed at top speed for 30 s, then centrifuged at 14,000g for 6 min at 4°C. For immunoblotting, lysates or immunoprecipitates were separated by SDS‐PAGE and proteins were transferred to nitrocellulose. Membranes were blocked with 5% milk in Tris‐buffered saline plus 0.05% Tween‐20 (TBST) and incubated with primary antibody overnight at 4°C. The next day, membranes were washed, incubated with appropriate horseradish peroxidase (HRP)‐coupled secondary antibodies, and signals were detected with ECL Western Blotting Substrate (Pierce, Rockford, IL, USA), ECL Plus Western Blotting Substrate (Pierce), or SuperSignal West Femto Maximum Sensitivity Substrate (Thermo Scientific, Waltham, MA, USA). The primary antibodies were phospho‐HDAC4/5/7 (S246/S259/S155) (Cell Signaling Technology, Beverly, MA, USA; 3443), total HDAC5 (AssayBiotech, Fremont, CA, USA; C0225), CRTC2 pS275,^(^ ^38^ ^)^ SIK2 pS358,^(^ ^24^ ^)^ and phospho‐PKA substrate (Cell Signaling Technology; 9624). Quantification was performed using ImageJ.

### 2.9 Subcellular fractionation

For subcellular fractionation, Ocy454 cells were initially resuspended in hypotonic lysis buffer (20mM HEPES, 10mM KCl, 1mM MgCl_2_, 0.1% Triton X‐100, 5% glycerol supplemented with protease inhibitor, 1mM NaF, 1mM DTT, and 1mM vanadate) for 5 min on ice. Nuclear pellets were spun down at 2300 × g for 5 min, and the supernatant was saved as the cytoplasmic lysate. Thereafter, the nuclear pellet was washed twice in 1 mL hypotonic lysis buffer. The nuclear pellet was resuspended in hypertonic lysis buffer (20mM HEPES, 400mM NaCl, 1mM EDTA, 0.1% Triton X‐100, 5% glycerol supplemented with protease inhibitor, 1mM NaF, 1mM DTT, and 1mM vanadate), followed by vortexing twice for 30 s. Debris was spun down at 16,100 × g for 5 min, and the supernatant was saved as the nuclear lysate.

### 2.10 Gene expression analysis

Total RNA was collected from cultured cells using QIAshredder (QIAGEN, Valencia, CA, USA) and PureLink RNA mini kit (Invitrogen, Carlsbad, CA, USA) following the manufacturer's instructions. Lysis buffer with 2‐mercaptoethanol was added to cold PBS washed cells and collected into QIAshredder, then centrifuged at 15,000*g* for 3 min. The flow‐through was collected into a new tube and RNA isolation was carried out with PureLink RNA mini kit. For qRT‐PCR, cDNA was prepared with 750 ng RNA using the Primescript RT kit (TaKaRa Bio, Otsu, Japan) and analyzed with PerfeCa® SYBR® Green FAstMix® ROX (QuantaBio, Beverly, MA, USA) in the StepOnePlus™ Real‐time PCR System (Applied Biosystems, Warrington, UK) using specific primers designed for each targeted gene. Relative expression was calculated using the delta‐delta comparative threshold cycle (2^–∆∆CT^) method by normalizing to β‐actin housekeeping gene expression, and presented as fold increase relative to β‐actin. Primers used were β‐actin (CCTCTATGCCAACACAGTGC and ACATCTGCTGGAAGGTGGAC), SOST (GCCTCATCTGCCTACTTGTG and CTGTGGCATCATTCCTGAAG), and RANKL (GCTGGGCCAAGATCTCTAAC and GTAGGTACGCTTCCCGATGT). Nanostring digital gene expression was performed using a custom probe set (Supplementary Table S1) according to the instructions of the manufacturer.

### 2.11 Statistical analyses

All statistical analyses were performed by GraphPad Prism 8 for Windows (GraphPad Software, Inc., La Jolla, CA, USA). Variables were tested by either two‐tailed *t* test or one‐way analysis of variance (ANOVA) followed by Tukey‐Kramer post hoc test. Values were expressed as mean ± SD unless otherwise stated. A *p* value <.05 was considered significant. The coefficiencies (*R* values) of cytofluorograms were calculated by ImageJ using JACoP with the representative images.

## 3 Results

### 3.1 Comparable effects of PTH analogs on intracellular calcium release

**Fig 1:**
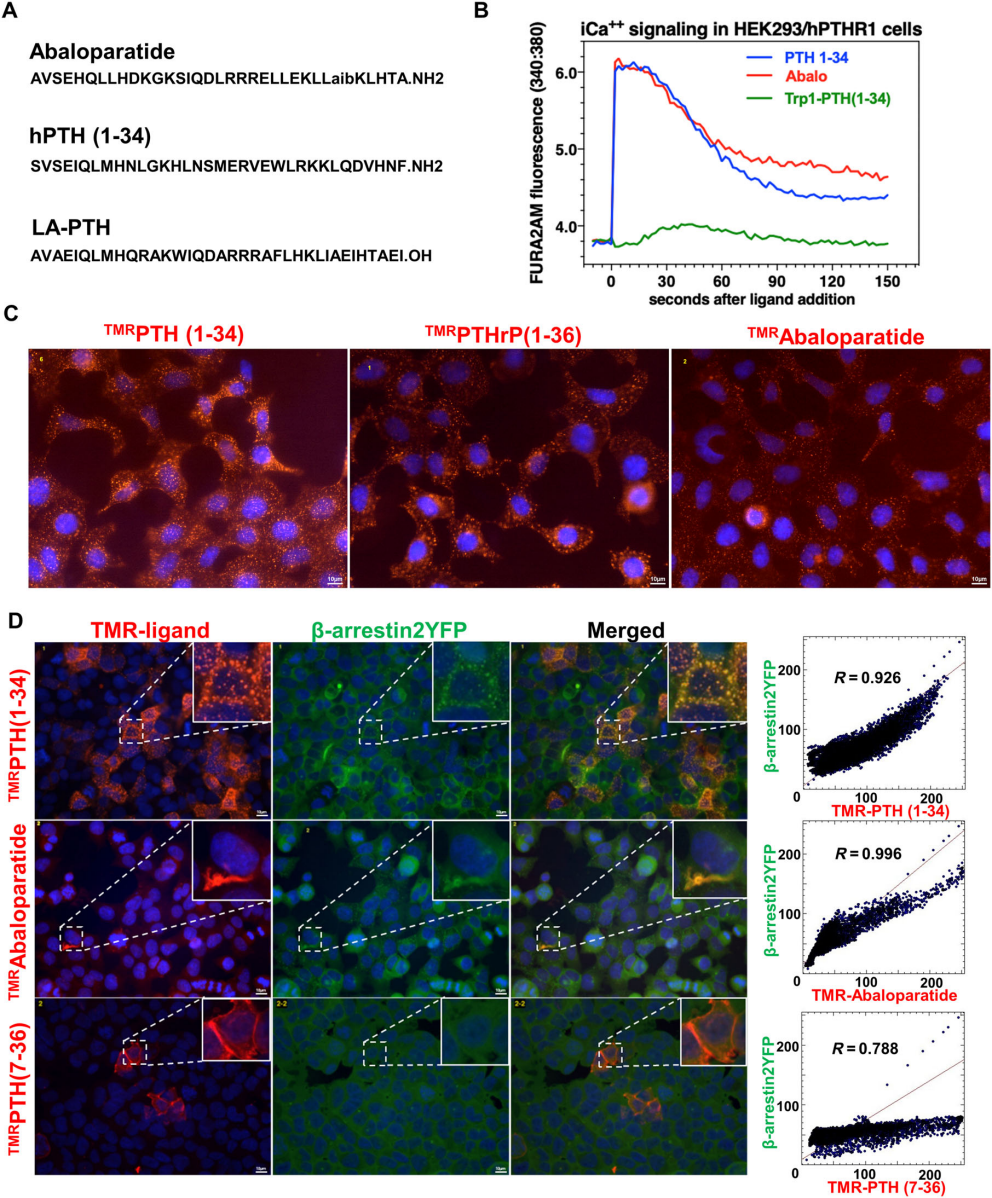
The effect of PTH analogs on intracellular calcium release and β‐arrestin recruitment. (*A*) The amino acid sequences of abaloparatide, human PTH (hPTH, teriparatide; TPTD) and long‐acting (LA)‐hPTH. (*B*) The intracellular calcium signaling was elevated by hPTH and abaloparatide. HEK293 cells (hPTH1R/glosensor (GP‐2.3) cells) were pretreated with calcium indicator (Fura2‐AM), and then treated with PTH analogs. The Trp1‐PTH (1‐34) was used as a negative control. (*C*) Tetramethyl‐rhodamine (TMR)‐labeled versions of PTH (^TMR^PTHc35 (1‐35), PTHrP (^TMR^PTHrP (1‐36)), and abaloparatide (^TMR^Abaloparatide). HEK293 cells stably transfected ratPTH1R/glosensor, GR‐35 cells were treated with PTH analogs for 15 min. All ligands induced ligand‐PTH1R internalization. Nuclei were stained with DAPI, as shown in blue. (*D*) The β‐arrestin clustering was induced by hPTH (1‐34) and abaloparatide. HEK293 cells stably expressing YFP‐tagged β‐arrestin2 were transiently transfected with human PTH1R (GBR‐24 cells), then treated with TMR‐labeled PTH ligands for 15 min. The cytofluorograms were generated by ImageJ using just another colocalization plugin (JACoP). The coefficiencies (*R* values) are calculated by JACoP with the representative images. There was no obvious difference between hPTH (1‐34) and abaloparatide. ^TMR^PTH (7‐36) was used as a negative control. Each experiment was repeated two times.

First, we assessed the ability of distinct PTH1R peptide ligands (Fig. 1*A*) to induce Gαq/PKC‐dependent increases in intracellular calcium in HEK293 cells stably expressing hPTH1R (GP‐2.3 cells). For this, cells were preloaded with the calcium indicator dye Fura2‐AM, and then treated with different PTH ligands at a concentration of 100nM. Intracellular calcium fluxes were measured by ratiometric fluorescence (excitation = 340 nm and 380 nm, emission = 535 nm) over time. As shown in Fig. 1*B*, both hPTH (1‐34) and abaloparatide stimulate the release of intracellular stored calcium to a comparable degree, whereas Trp1‐PTH (1‐34) (a PTH analog defective at activating Gαq/phospholipase signaling^(^ ^32^ ^)^) does not stimulate intracellular calcium in this system.

### 3.2 Comparable effects of PTH analogs on PTH1R internalization

Next, we asked whether PTH analogs might have different effects on PTH1R activation‐dependent internalization. For this, HEK293 cells stably expressing a rat PTH1R cDNA (GR‐35 cells) were treated with tetramethyl‐rhodamine‐labeled versions of hPTH (1‐34), PTHrP, and abaloparatide (the TMR fluorophore is attached at the K13 position of each peptide). Cells were treated for 15 min at room temperature (21°C) at peptide doses of 10nM. After excess unbound peptide was washed away, cells were examined by epifluorescent microscopy. As shown in Fig. 1*C*, all ligands exhibit clustering in these conditions, indicating that all ligands are effectively internalized.

### 3.3 Comparable effects of PTH analogs on β‐arrestin recruitment

Recruitment of the intracellular scaffolding proteins β‐arrrestin1/2 is a well‐characterized step that occurs following activation and GRK‐dependent phosphorylation in multiple G‐Protein‐Coupled Receptors (GPCRs) signaling systems.^(^ ^39^ ^)^ β‐arrestin recruitment is traditionally thought to lead to signal termination due to receptor internalization. However, work over the past decade, stimulated by detailed studies of intracellular PTH1R signaling,^(^ ^8^ ^)^ has demonstrated that β‐arrestin recruitment promotes GPCR signaling via cAMP that occur in endosomes.^(^ ^40^ ^)^ Therefore, we assessed β‐arrestin recruitment by distinct PTH1R ligands. For this, HEK293 cells stably expressing YFP‐tagged β‐arrestin2 (GBR‐24 cells) were transiently transfected with human PTH1R cDNA, then treated with TMR‐labeled PTH ligands at 30nM for 15 min at room temperature. Thereafter, cells were rinsed, fixed, and viewed by epifluorescent microscopy. As shown in Fig. 1*D*, although all ligands bind PTH1R, only hPTH (1‐34) and abaloparatide induce β‐arrestin2 clustering. Here, PTH (7‐36), a peptide that does not induce intracellular signaling or PTH1R internalization,^(^ ^41^ ^)^ fails to stimulate β‐arrestin2 recruitment. In these assays, no differences were noted in the extent of β‐arrestin2 clustering comparing hPTH (1‐34) and abaloparatide.

**Fig 2:**
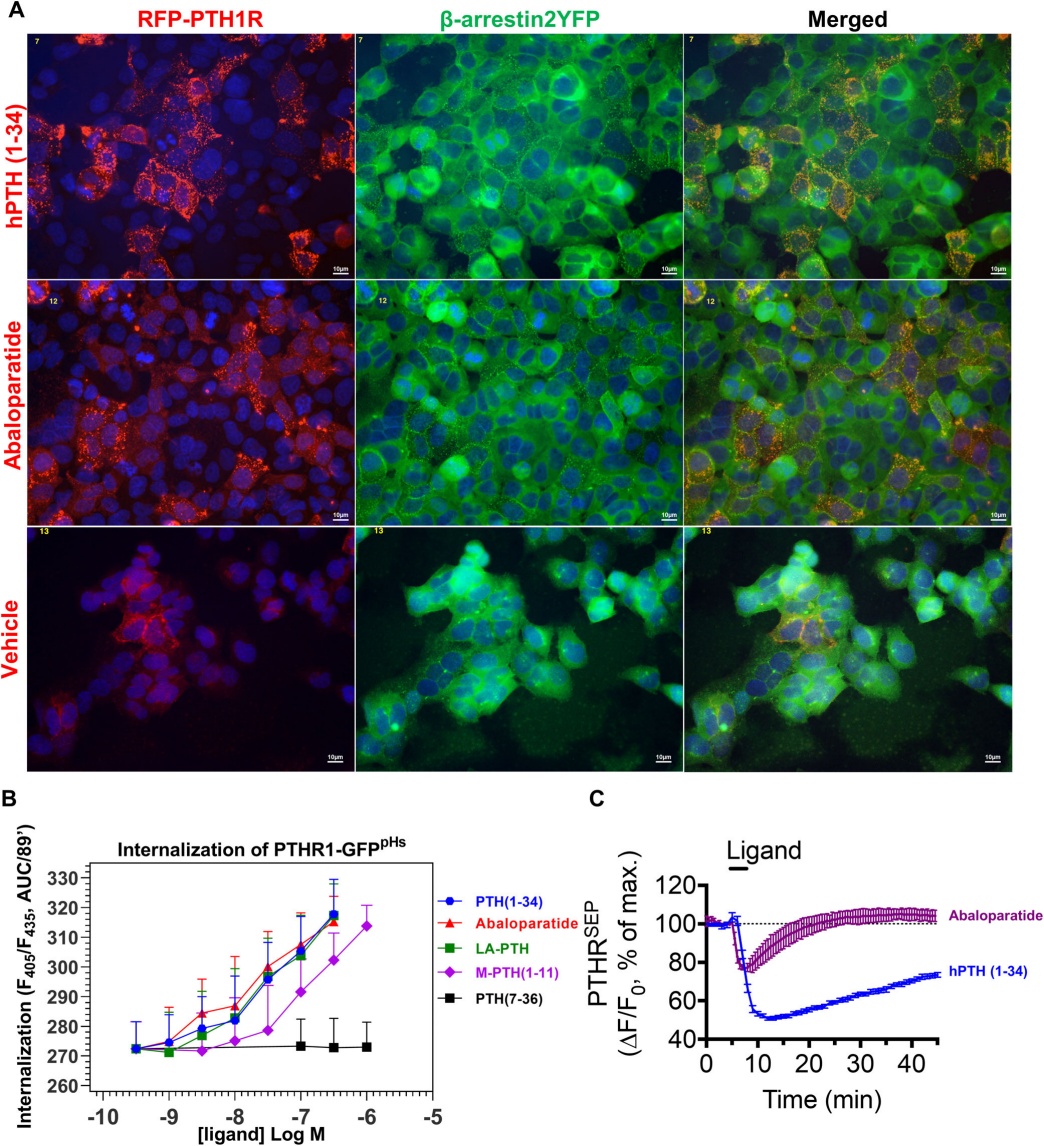
The effect of PTH analogs on β‐arrestin recruitment and PTH1R internalization. (*A*) The β‐arrestin2‐YFP‐expressing HEK293 cells (GBR‐24 cells) were transiently transfected with a DsRed‐tagged human PTH1R (RFP‐PTH1R shown in red). After that, cells were treated with hPTH (1‐34) or abaloparatide for 15 min. hPTH (1‐34) and abaloparatide induced the hPTH1R/β‐arrestin2 colocalization in hPTH1R expressing cells. There was no difference between hPTH (1‐34) and abaloparatide. PTH1R (red), β‐arrestin2‐YFP (green), and DAPI (blue). (*B*) HEK293 cells stably expressing PTH1R‐GFP^pHs^ (GPG‐10 cells) were treated continuously with the indicated doses of PTH ligands for 90 min. Internalization was measured as the AUC of the GFP^pHs^ fluorescence ratio from *n* = 6 independent experiments. (*C*) PTH1R‐GFP^SEP^ expressing HEK293 cells (stably expressing the pH‐sensitive GFP super‐ecliptic pHluorin (SEP)‐PTH1R) were treated for 5 min with the indicated peptide (100nM) followed by kinetic washout. PTH1R‐GFP^SEP^ internalization and disappearance from acidic subcellular compartments was measured over the subsequent 25 min. hPTH (1‐34) and abaloparatide induced the PTH1R internalization after “washout” procedure. However, abaloparatide showed significantly quicker PTH1R recycling from the cytosol components to the plasma membrane than hPTH (1‐34). Data are expressed as mean ± SD. Each experiment was repeated two times.

A complementary approach was used to visualize ligand‐dependent colocalization of PTH1R and β‐arrestin2. For this, β‐arrestin2‐YFP–expressing cells were transiently transduced with a plasmid encoding a dsRed‐tagged version of human PTH1R. Thereafter, cells were treated with hPTH (1‐34) and abaloparatide (30nM) for 15 min at room temperature (21°C) and PTH1R/β‐arrestin colocalization was visualized by epifluorescent microscopy. As shown in Fig. 2*A*, hPTH (1‐34) and abaloparatide both induce PTH1R/β‐arrestin2 colocalization. In sum, these studies in PTH1R‐expressing HEK293 cells indicate that hPTH (1‐34) and abaloparatide both induce calcium signaling, receptor internalization, and β‐arrestin2 recruitment to a comparable degree. These results, obtained at a single ligand concentration and at the single time point of 30 min after ligand addition, differ from those of a previous Bioluminescence Resonance Energy Transfer (BRET)‐based kinetic study in which abaloparatide exhibited a moderately weaker potency than hPTH (1‐34) for inducing the interaction of PTH1R and β‐arrestin. In that study, multiple ligand concentrations were used and the responses were assessed at the peak response time, which was generally ~5 min after ligand addition.^(^ ^42^ ^)^ To explore differences in receptor internalization in more detail, we used constructs where the PTH receptor is fused to a pH‐sensitive GFP variant (GFP^pHs^) which changes its fluorescent signal in acidic endosomes. As shown in Fig. 2*B*, across a range of different doses, no obvious differences were noted in internalization between hPTH (1‐34), abaloparatide, and LA‐PTH in short‐term studies examining the “on” kinetics of this response. Here, the N‐terminally truncated PTH (7‐36) antagonist peptide was again used as an internalization‐deficient PTH1R ligand, and the C‐terminally truncated M‐PTH (1‐11) agonist peptide^(^ ^43^ ^)^ was used as an additional control. In contrast, kinetic washout studies (Fig. 2*C*) demonstrated that, although both abaloparatide and hPTH (1‐34) drive comparable initial PTH1R redistribution into acidic subcellular compartments, after washout, hPTH (1‐34) shows sustained internalization versus abaloparatide.

### 3.4 Comparable effects of PTH analogs on cAMP generation and PKA activation in Ocy454 cells

Next, we sought to define the effects of distinct PTH1R ligands on endogenous receptors in Ocy454 cells. This is a conditionally‐immortalized murine osteocyte‐like cell line derived from bi‐transgenic mice expressing DMP1‐GFP^topaz^ and a temperature sensitive large T antigen.^(^ ^30^, ^31^ ^)^ For these studies, a single cell subclone of the parental Ocy454 cell population (termed clone 6‐9) was used. For biochemical endpoints, cells were grown at the nonpermissive growth condition (37°C) for 7 days. For gene expression endpoints, cells were grown at the nonpermissive growth condition (37°C) for 14 days. In general, our approach was to “walk down” the PTH1R signaling pathway using well‐established assays to determine if signaling differences may exist between different PTH1R ligands.

**Fig 3:**
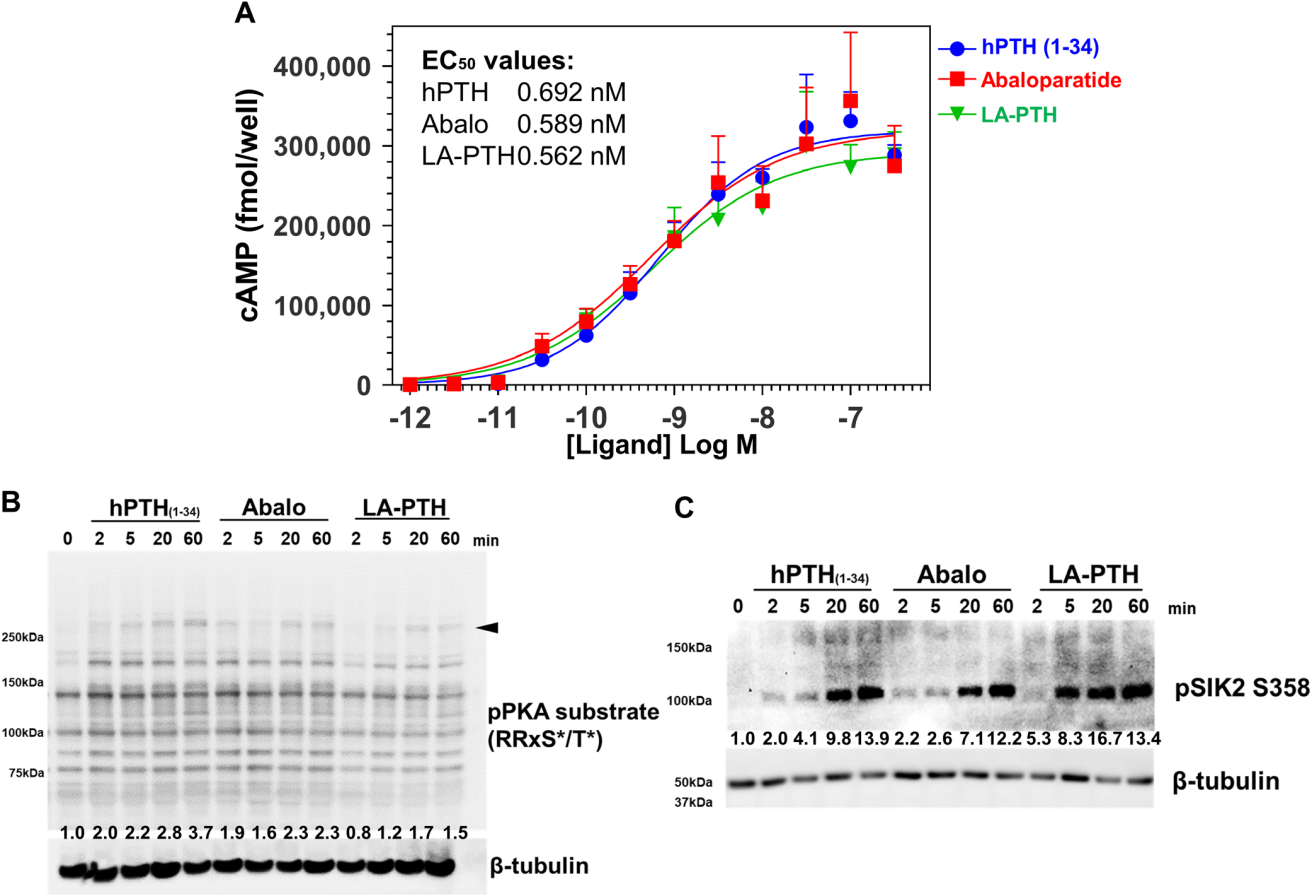
The effect of PTH analogs on cAMP generation and PKA activation in Ocy454 cells. (*A*) Ocy454 cells, a mouse osteocytic cell line were treated with different doses of peptides in the presence of IBMX for 30 min at room temperature, followed by radioimmunoassay for cAMP. (*B*) Western blotting of cAMP‐dependent PKA substrates. Ocy454 cells were treated PTH analogs (3.6nM) for different time points. All PTH analogs induced rapid phosphorylation of multiple cellular proteins (one such high molecular weight band denoted by arrowhead is quantified below the immunoblot) bearing the RRxS*/T* motif. (*C*) PTH analogs induced the SIK2 S358 phosphorylation. All PTH analogs induced rapid phosphorylation of SIK2 on S358. Data are expressed as mean ± SD. Each experiment was repeated two times (*B*, *C*) or three times (*A*). IBMX = 3‐isobutyl‐1‐methylxanthine; PKA = protein kinase A.

First, we assessed the effects of different PTH analogs on cAMP generation. For this, cells were treated with different concentrations of peptides in the presence of IBMX for 30 min at room temperature, followed by radioimmunoassay for cAMP. As shown in Fig. 3*A*, comparable cAMP generation curves were noted between the three PTH1R peptide agonists tested.

A major cellular effect of cAMP is to activate cAMP‐dependent PKA.^(^ ^44^ ^)^ Therefore, we next measured PKA activity in Ocy454 cells treated with different PTH peptides using a degenerate phospho‐specific antibody that recognizes proteins phosphorylated at a consensus PKA site (RRxS*/T*). For this, cells were treated with hPTH (1‐34), abaloparatide, or LA‐PTH for different time points at a concentration of 3.6nM. A dose close to the median effective dose (EC_50_, half maximal effective concentration) of all peptides was chosen to increase potential sensitivity to detect differences between PTH1R agonist activity. As shown in Fig. 3*B*, all ligands induce rapid phosphorylation of multiple cellular proteins bearing the RRxS*/T* consensus PKA phosphorylation motif. Consistent with the cAMP data above, no obvious differences were noted between the effects of each PTH1R agonist with respect to this biochemical endpoint.

### 3.5 Comparable effects of PTH analogs on engagement of the SIK pathway

Recent work has shown that a key step linking PKA activation to downstream changes in cellular gene expression is PKA‐dependent phosphorylation and inhibition of salt inducible kinases (for review^(^ ^26^, ^45^ ^)^). SIKs are AMPK family kinases whose cellular activity is potently inhibited by direct PKA‐mediated phosphorylation through an allosteric mechanism involving recruitment of 14‐3‐3 binding proteins.^(^ ^46^ ^)^ We recently reported that PTH signaling in osteocytes and osteoblasts leads to SIK inhibition, both in vitro^(^ ^24^ ^)^ and in vivo.^(^ ^27^ ^)^ PKA phosphorylates SIK2 at multiple sites including serine 358.^(^ ^47^ ^)^ As shown in Fig. 3*C*, no differences in SIK2 S358 phosphorylation were noted across the different PTH peptides that were tested.

**Fig 4:**
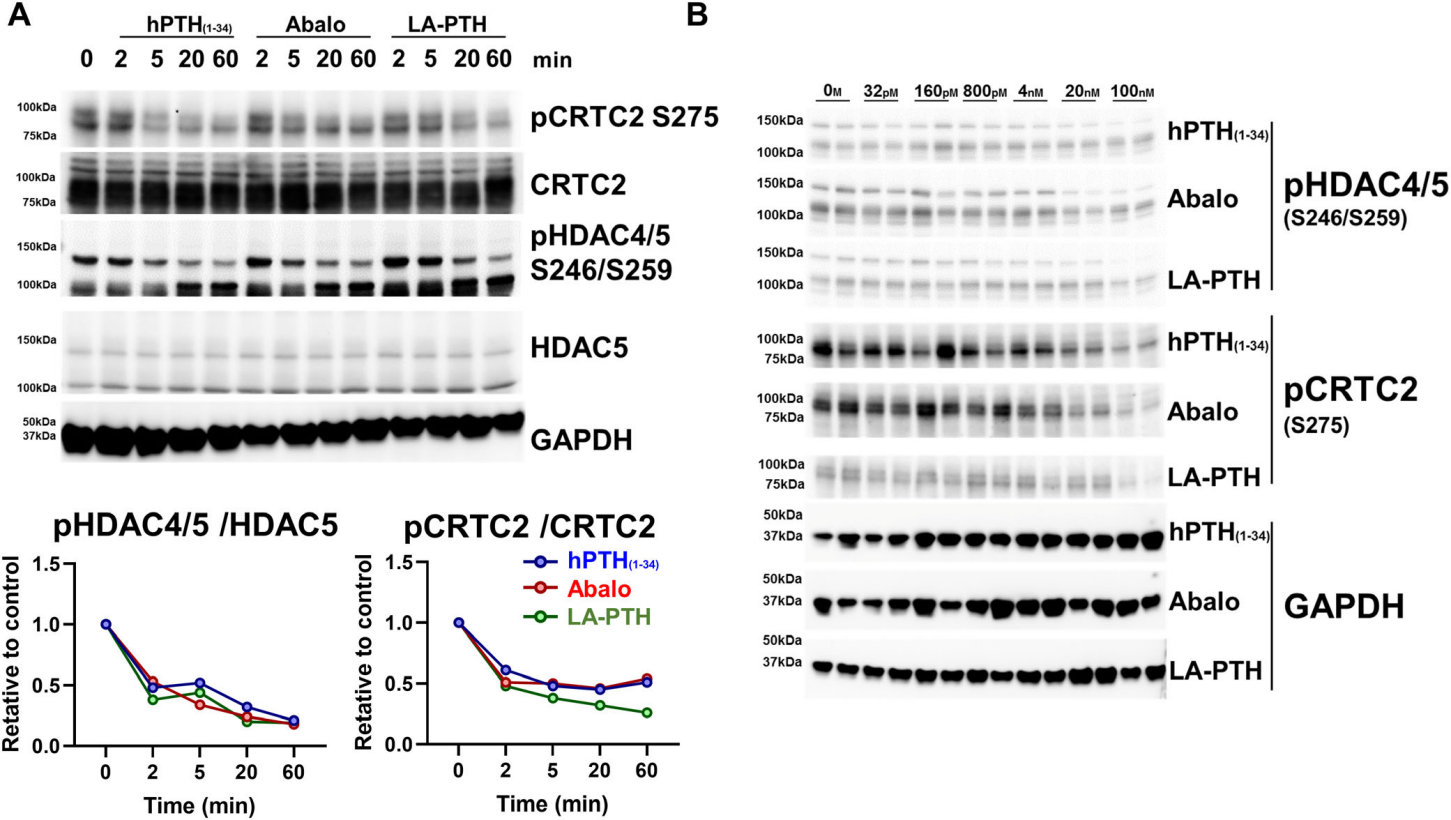
The effect of PTH analogs on SIK substrate (phospho‐CRTC2 and phospho‐HDAC4/5) phosphorylation. (*A*) Ocy454 cells, a mouse osteocytic cell line were grown at 37°C for 7 days, then treated with 3.6nM of PTH analogs for the indicated times followed by immunoblotting. Densitometry was performed to assess HDAC4/5 and CRTC2 phosphorylation as normalized to the each baseline at 0 min. No obvious differences were noted among PTH analogs with respect to reducing CRTC2, HDAC4, and HDAC5 phosphorylation. (*B*) The dose responses of PTH analogs. The PTH analogs were treated for 60 min with the indicated doses, followed by immunoblotting. No obvious differences were noted among the PTH analogs with respect to reducing CRTC2, HDAC4, and HDAC5 phosphorylation. Each experiment was repeated three times (*A*) or two times (*B*).

**Fig 5:**
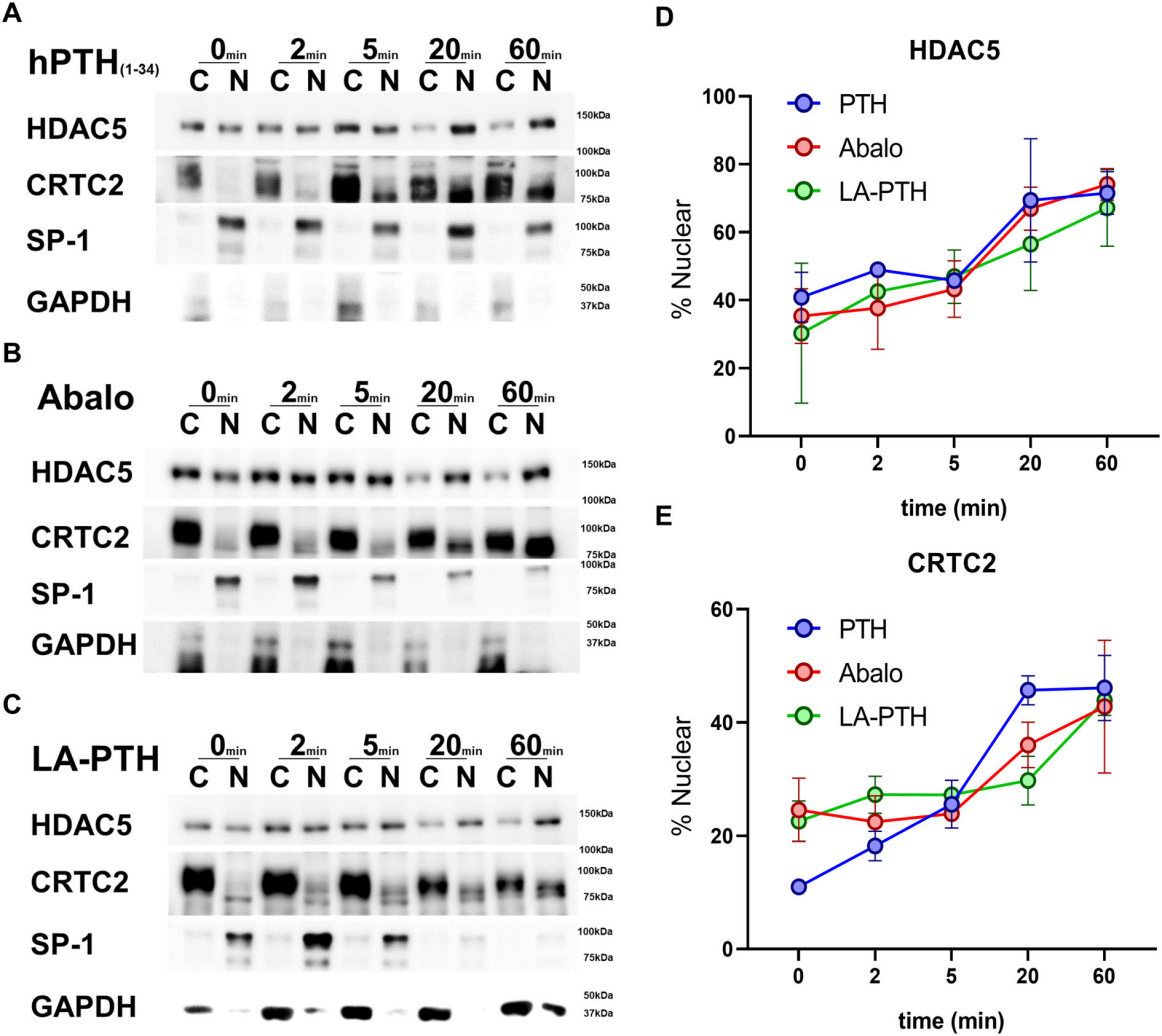
The effect of PTH analogs on SIK substrate (HDAC5 and CRTC2) subcellular localization. Ocy454 cells, a mouse osteocytic cell line were treated with 10nM (*A*) hPTH (1‐34), (*B*) abaloparatide and (*C*) LA‐PTH for the indicated times and subjected to subcellular fractionation followed by immunoblotting. Biologic duplicates per time point were studied. The nuclear fraction of endogenous HDAC5 and CRTC2 was quantified by densitometry using ImageJ at each time point. No obvious differences were noted among the PTH analogs with respect to increasing HDAC5 and CRTC2 nuclear translocation. Data are expressed as mean ± SD. Each experiment was repeated two times.

In cells, SIKs are constitutively active kinases due to upstream LKB1 activation loop phosphorylation,^(^ ^45^ ^)^ and therefore constitutively phosphorylate their substrates. The best‐described SIK substrates are class IIa HDACs and CRTC proteins.^(^ ^26^ ^)^ Both class IIa HDACs and CRTC are retained in the cytoplasm when phosphorylated by SIKs, and translocate into the nucleus upon dephosphorylation when cellular SIK activity is inhibited by cAMP/PKA signaling. PTH and small molecule SIK inhibitors reduce class IIa HDAC and CRTC phosphorylation in cells and promote the subsequent nuclear translocation of these factors.^(^ ^24^ ^)^ In the nucleus, CRTC proteins stimulate RANKL expression whereas class IIa HDAC proteins suppress sclerostin expression. We assessed the effects of different PTH ligands on class IIa HDAC and CRTC phosphorylation at well‐documented SIK phosphorylation sites: HDAC4 S246, HDAC5 S259, and CRTC2 S275. As shown in Fig. 4*A*,*B*, no obvious differences were noted between the ability of each PTH ligand to reduce phosphorylation levels of these SIK substrates. The dephosphorylation of SIK substrates induces their translocation from cytoplasm to nuclei. We analyzed the subcellular localization of SIK substrates (HDAC5 and CRTC2) over time in response to treatment with distinct PTH1R ligands. All PTH analogs promoted HDAC5 and CRTC2 translocation from cytoplasm to nucleus within 60 min. There were no significant differences among the PTH analogs (Fig. 5 *A‐E*). Taken together, these data indicate that all three PTH analogs tested (hPTH (1‐34), abaloparatide, and LA‐PTH) engage the cAMP/PKA/SIK pathway to a comparable degree.

### 3.6 Comparable effects of PTH analogs on target gene regulation

**Fig 6:**
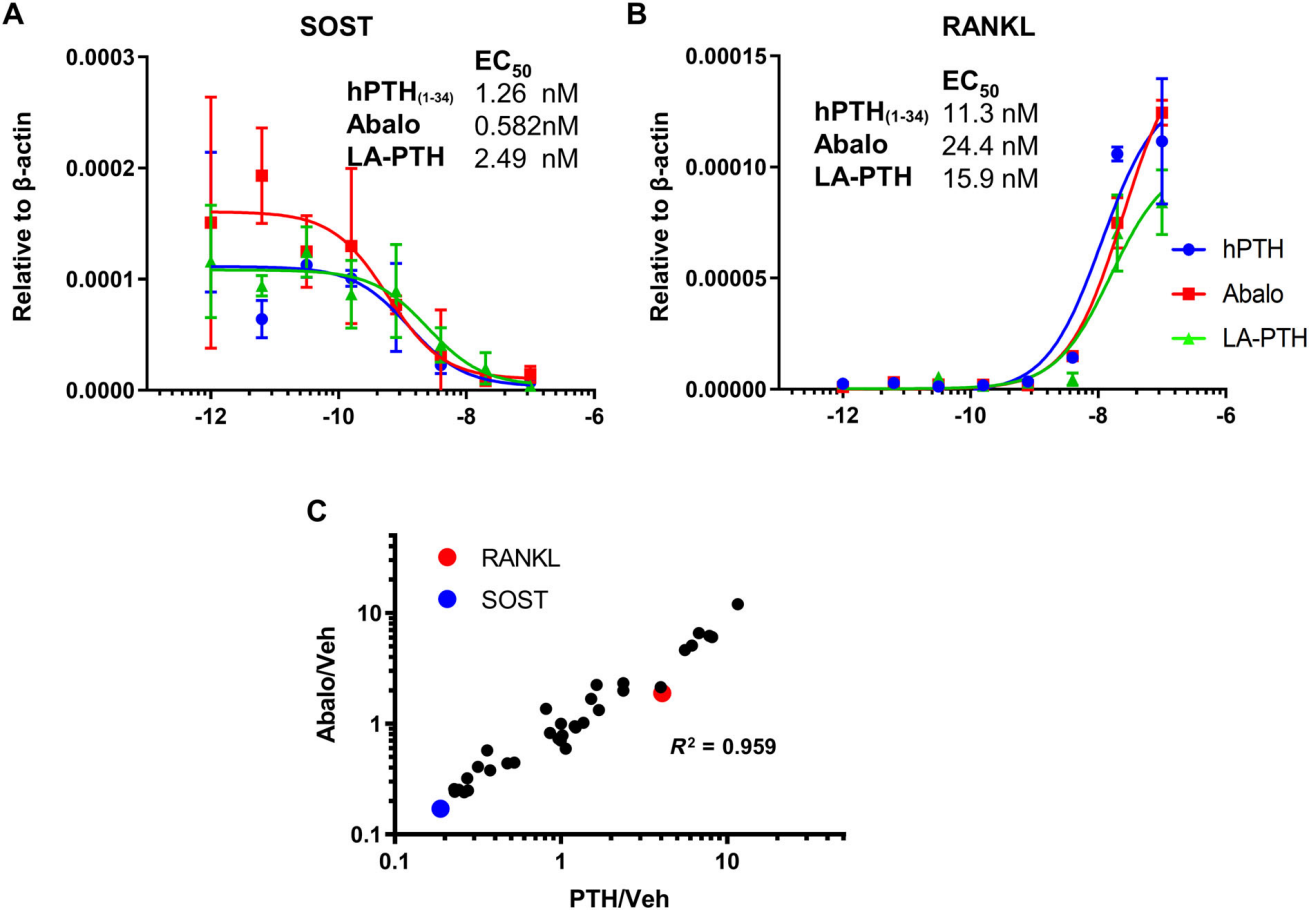
The effect of PTH analogs on gene expression in osteocytes. Ocy454 cells, a mouse osteocytic cell line were grown at 37°C for 14 days, then treated with the indicated peptides for 4 hours followed by RT‐qPCR. No differences were noted with respect to transcriptional regulation of SOST (*A*) and RANKL (*B*). (*C*) Ocy454 cells were treated with hPTH (1‐34) and abaloparatide (20nM) for 4 hours followed by RNA isolation. The expression of 32 PTH/SIK target genes were assessed by Nanostring profiling. The fold change of each gene induced by hPTH (1‐34) or abaloparatide versus vehicle is shown on this scatterplot. All genes are comparably regulated by each peptide. Data are expressed as mean ± SD. Each experiment was repeated two times.

Changes in SIK substrate phosphorylation result in nuclear translocation and target gene regulation. Two well‐characterized PTH target genes in osteocytes are SOST (encodes sclerostin) and TNFSF11 (encodes RANKL). PTH‐mediated SOST suppression contributes to bone anabolism, while PTH‐mediated RANKL induction stimulates bone resorption.^(^ ^22^ ^)^ Therefore, we performed RT‐qPCR to measure the ability of PTH ligands to regulate these target genes. As predicted by our biochemical studies (Fig. 3‐5), all three ligands led to comparable dose‐dependent regulation of SOST and RANKL (Fig. 6*A*,*B*). PTH‐dependent SIK substrate phosphorylation and gene expression changes depend upon receptor activation and internalization, as evidenced by the observation that PTH (7‐36) is completely inactive with respect to these endpoints (Supplementary Fig. S1).

Expression of many additional genes are regulated by the PTH/SIK pathway in target cells in bone. We have developed a custom Nanostring digital gene expression (DGE) profiling target set of 32 PTH/SIK‐regulated genes, based on our previous RNAseq data comparing the effects of PTH and SIK inhibitors in Ocy454 cells.^(^ ^24^ ^)^ RNA samples from cells treated with 20nM of hPTH (1‐34) and abaloparatide from Fig. 6*A*,*B* were analyzed using this custom probe set. As shown in Fig. 6 *C* and Supplementary Figs. S2 and S3, concordant effects (*R* ^2^ = 0.96) were noted comparing hPTH (1‐34) and abaloparatide across the set of PTH1R‐regulated osteocyte target genes. In the scatterplot in Fig. 6*C*, each data point represents an individual PTH1R‐regulated target gene. The position of the data point on the *x* axis represents the average fold change for that gene in response to PTH (compared to vehicle control), whereas the position of the data point on the y‐axis represents the average fold change for that gene in response to abaloparatide.

## 4 Discussion

The goal of this study was to determine if differences exist between distinct PTH analogs at the level of intracellular signaling. As shown above, our data demonstrate mostly comparable effects of hPTH (1‐34), abaloparatide, and LA‐PTH were noted in these short‐term, single‐dose signaling studies. Hattersley and colleagues^(^ ^5^ ^)^ demonstrated that abaloparatide shows differential binding selectivity and signaling duration versus hPTH (1‐34) using heterologous expression systems. Additionally, other groups have also directly compared abaloparatide and hPTH (1‐34) in similar (but not identical) in vitro and in vivo experimental systems.^(^ ^48^, ^49^, ^50^, ^51^ ^)^ Although subtle differences have been reported between hPTH (1‐34) and abaloparatide, overall, the biologic actions of these peptides appear qualitatively quite similar when compared side‐by‐side in cell culture systems. Here, it is important to note that the previous differences between hPTH (1‐34), abaloparatide, and LA‐PTH have mainly been observed in kinetic “washout” protocols that rely upon rapid and repeated measurement of signals in plate‐reader assays and/or highly‐sensitive single cell measurements by Fluorescence Resonance Energy Transfer (FRET)‐based methods.^(^ ^52^ ^)^ The same general principal applies for multiple GPCR signaling systems where important biologic differences in kinetic signaling output have been reported.^(^ ^53^ ^)^ We confirmed a faster washout profile for abaloparatide versus PTH (1‐34) in a single‐cell kinetic assay of receptor recycling. Current assays to interrogate the downstream signaling changes under investigation here—inhibitory SIK phosphorylation, SIK substrate dephosphorylation, and subsequent gene expression changes—are insufficiently robust to employ a similar kinetic washout study design.

A major goal of this study was to determine the effects of different PTH analogs on osteocyte gene expression because it is well‐established that osteocytes are a major cell type targeted by PTH in vivo.^(^ ^19^, ^23^, ^54^, ^55^ ^)^ Unfortunately, reproducible methods for primary cell culture of osteocytes are not possible; for this reason we have focused primarily on the well‐described Ocy454 cell line^(^ ^30^ ^)^ as a reliable model system to study PTH‐induced signaling and gene expression changes in osteocytes. Currently, no human osteocyte‐like cell lines are available for signaling and gene expression studies. That being said, PTH and its receptor are well‐conserved across species, and extensive previous studies (reviewed in^(^ ^56^ ^)^) have revealed potent effects of human PTH 1‐34 on the murine PTH receptor.

It is possible that differences observed in vivo in preclinical and clinical models may be related to pharmacokinetic factors, although the serum half life reported for the different peptides are quite similar.^(^ ^11^, ^57^, ^58^ ^)^ It is also possible that our current in vitro systems are insufficient to perfectly match the complexities of PTH1R signaling in bona fide target cells in bone in vivo. Related to this is the possibility that PTH1R‐expressing target cells distinct from osteocytes may be responsible for biologic differences observed between hPTH (1‐34), abaloparatide, and LA‐PTH.

## Disclosures

MNW and TJG received research funding from Radius Health, Inc. MNW receives research funding from Galapagos NV. The remaining authors declare no competing interests.

## AUTHOR CONTRIBUTIONS

Tadatoshi Sato: Conceptualization; data curation; formal analysis; investigation; methodology; writing‐original draft; writing review and editing. Shiv Verma: Data curation; formal analysis; investigation. Ashok Khatri: Investigation; methodology; formal analysis. Thomas Dean: Data curation; formal analysis; investigation. Olga Goransson: Methodology; writing review and editing. Thomas J Gardella: Conceptualization; data curation; formal analysis; investigation; methodology; writing‐original draft; writing review and editing. Marc N Wein: Conceptualization; data curation; formal analysis; investigation; methodology; writing‐original draft; writing review and editing.

## Author's roles

TS, SV, AK, TD, and TJG performed experiments. TS, SV, TD, TJG, and MNW analyzed the data. TS and MNW wrote the manuscript. All authors reviewed and approved the manuscript.

5

### Peer Review

The peer review history for this article is available at https://publons.com/publon/10.1002/jbm4.10441.

## Supporting information


**Supplementary Fig. S1 Effects of hPTH(1‐34) and PTH (7‐36) in Ocy454 cells.** (A) Ocy454 cells were treated with 10nM PTH analogs (hPTH (1‐34), PTH (7‐36)) for 4 hours. As expected, hPTH (1‐34) reduced Sost and increased Rankl expression. However, PTH (7‐36) did not affect expression of these PTH‐regulated genes. (*n* = 4 biologic replicates) *p* values versus control, ****p < .001. One‐way ANOVA followed by Tukey‐Kramer post hoc test was used. Data are expressed as mean ± SD. (8) Western blotting was performed with pHDAC4/5 (S246/S255) or total HDAC5 antibodies. Ocy454 cells were treated with 10nM hPTH (1‐34) or 10nM PTH (7‐36) for 1 hour followed by immunoblotting. Only hPTH (1‐34) significantly decreased pHDAC4/5 levels. r3‐tubulin is used as a loading control.
**Supplementary Fig. S2**: Ocy454 cells were treated with 20nM PTH analogs (hPTH (1‐34), ABL and LA‐PTH) for 4 hours. The expression of 32 PTH/SIK target genes were assessed by Nanostring using nCounter system using nSolver software. Each gene expression was normalize by r3‐actin. The heatmap was generated by GraphPad Prism 8.4 software. All PTH analogs showed similar expression pattern by 4 hours of treatment and clear induction of gene changes compared with the vehicle treatments.
**Supplementary Fig. S3**: Ocy454 cells were treated with 20nM PTH analogs (hPTH (1‐34), ABL and LA‐PTH) for 4 hours. The expression of 32 PTH/SIK target genes were assessed by Nanostring. The expression was normalized by the house keeping gene r3‐actin. Most gene expression changes showed no obvious differences among the PTH analogs tested. Data are expressed as mean ± SD of two biologic replicates.

Supplementary Table S1
