# Supplementary material for: Comparable Initial Engagement of Intracellular Signaling Pathways by Parathyroid Hormone Receptor Ligands Teriparatide, Abaloparatide, and Long‐Acting PTH
Source: JBMR Plus. 2021 May 6;5(5):e10441. doi: 10.1002/jbm4.10441 (PMC8101618; doi:10.1002/jbm4.10441)
Supplement: Supplementary file 1 — Supplementary Fig. S1 Effects of hPTH(1‐34) and PTH (7‐36) in Ocy454 cells. (A) Ocy454 cells were treated with 10nM PTH analogs (hPTH (1‐34), PTH (7‐36)) for 4 hours. As expected, hPTH (1‐34) reduced Sost and increased Rankl expression. However, PTH (7‐36) did not affect expression of these PTH‐regulated genes. (n = 4 biologic replicates) p values versus control, ****p < .001. One‐way ANOVA followed by Tukey‐Kramer post hoc test was used. Data are expressed as mean ± SD. (8) Western blotting was performed with pHDAC4/5 (S246/S255) or total HDAC5 antibodies. Ocy454 cells were treated with 10nM hPTH (1‐34) or 10nM PTH (7‐36) for 1 hour followed by immunoblotting. Only hPTH (1‐34) significantly decreased pHDAC4/5 levels. r3‐tubulin is used as a loading control. Supplementary Fig. S2: Ocy454 cells were treated with 20nM PTH analogs (hPTH (1‐34), ABL and LA‐PTH) for 4 hours. The expression of 32 PTH/SIK target genes were assessed by Nanostring using nCounter system using nSolver software. Each gene expression was normalize by r3‐actin. The heatmap was generated by GraphPad Prism 8.4 software. All PTH analogs showed similar expression pattern by 4 hours of treatment and clear induction of gene changes compared with the vehicle treatments. Supplementary Fig. S3: Ocy454 cells were treated with 20nM PTH analogs (hPTH (1‐34), ABL and LA‐PTH) for 4 hours. The expression of 32 PTH/SIK target genes were assessed by Nanostring. The expression was normalized by the house keeping gene r3‐actin. Most gene expression changes showed no obvious differences among the PTH analogs tested. Data are expressed as mean ± SD of two biologic replicates. [file JBM4-5-e10441-s002.pdf]

Supplementary figure 1

A

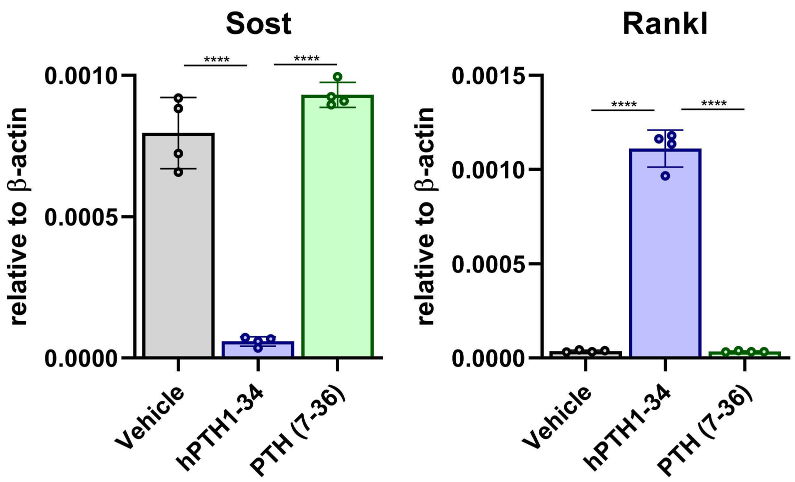


B

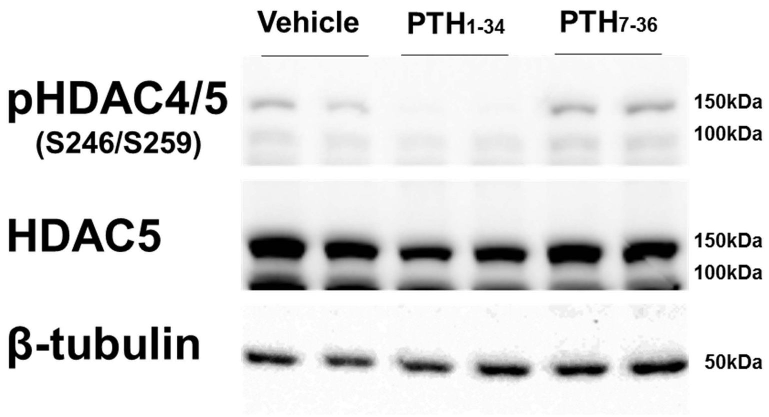

**Supplementary Figure 1: Effects of hPTH(1-34) and PTH (7-36) in Ocy454 cells.** (A) Ocy454 cells were treated with 10 nM PTH analogs (hPTH (1-34), PTH (7-36)) for 4 hours. As expected, hPTH(1-34) reduced Sost and increased Rankl expression. However, PTH (7-36) did not affect expression of these PTH-regulated genes. (n = 4 biologic replicates) P-values vs control, **** $p<0.001$. One-way ANOVA followed by Tukey–Kramer post hoc test was used. Data are expressed as mean ± SD. (B) Western blotting were performed with pHDAC4/5 (S246/S255) or total HDAC5 antibodies. Ocy454 cells were treated with 10 nM hPTH (1-34) or 10 nM PTH (7-36) for 1 hour followed by immunoblotting. Only hPTH (1-34) significantly decreased pHDAC4/5 levels. β-tubulin is used as a loading control.

**Supplementary figure 2**

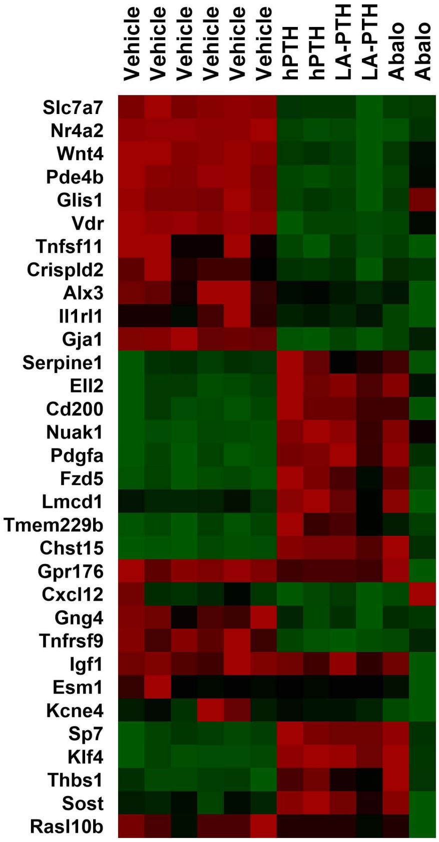

**Supplementary Figure 2:**
Ocy454 cells were treated with 20 nM PTH analogs (hPTH (1-34), ABL and LA-PTH) for 4 hours. The expression of 32 PTH/SIK target genes were assessed by Nanostring using nCounter system using nSolver software. Each gene expression was normalize by β-actin. The heatmap was generated by GraphPad Prism 8.4 software. All PTH analogs showed similar expression pattern by 4h treatment and clear induction of gene changes compared with the vehicle treatments.

**Supplementary figure 3**

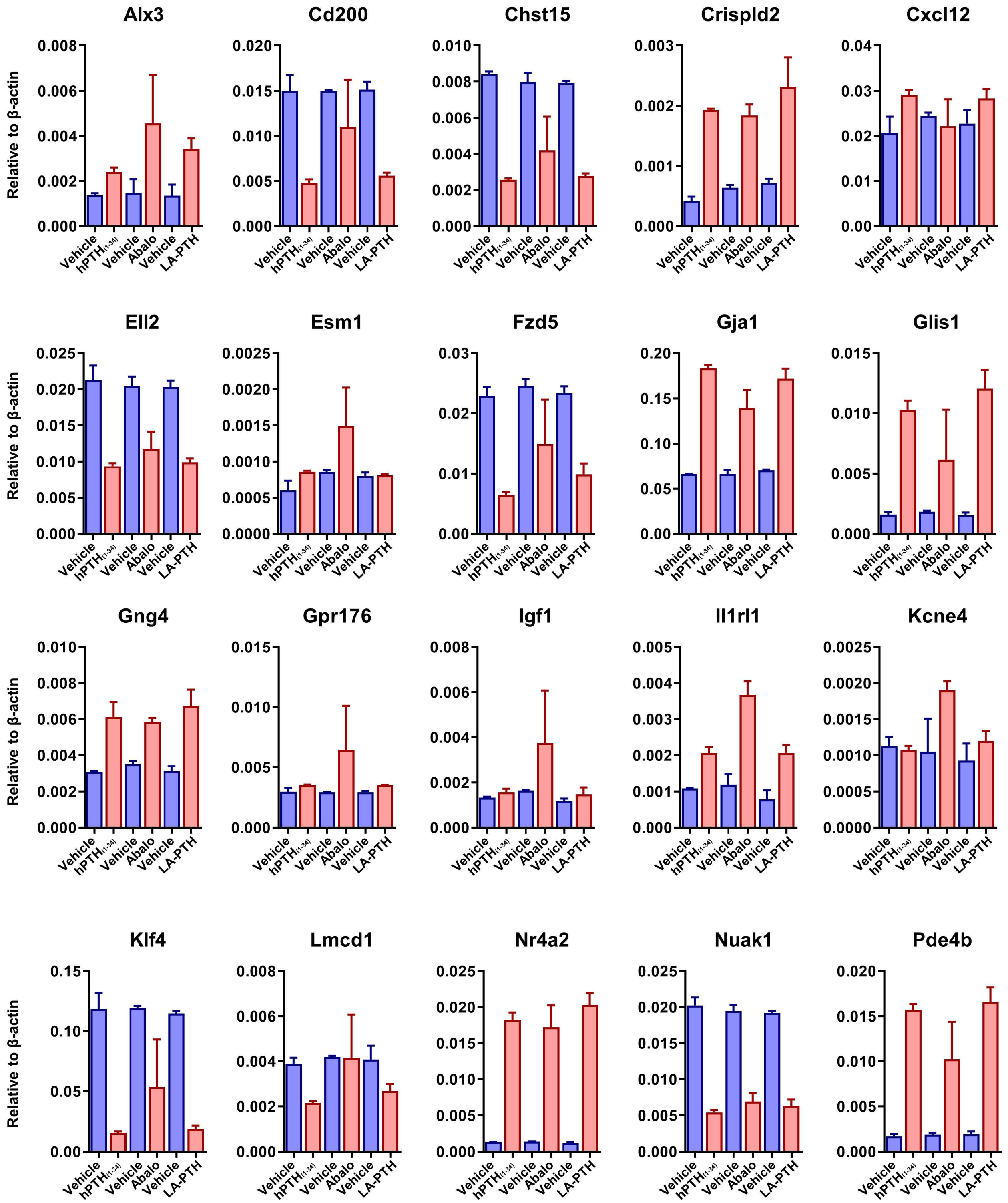

Supplementary figure 3 continued

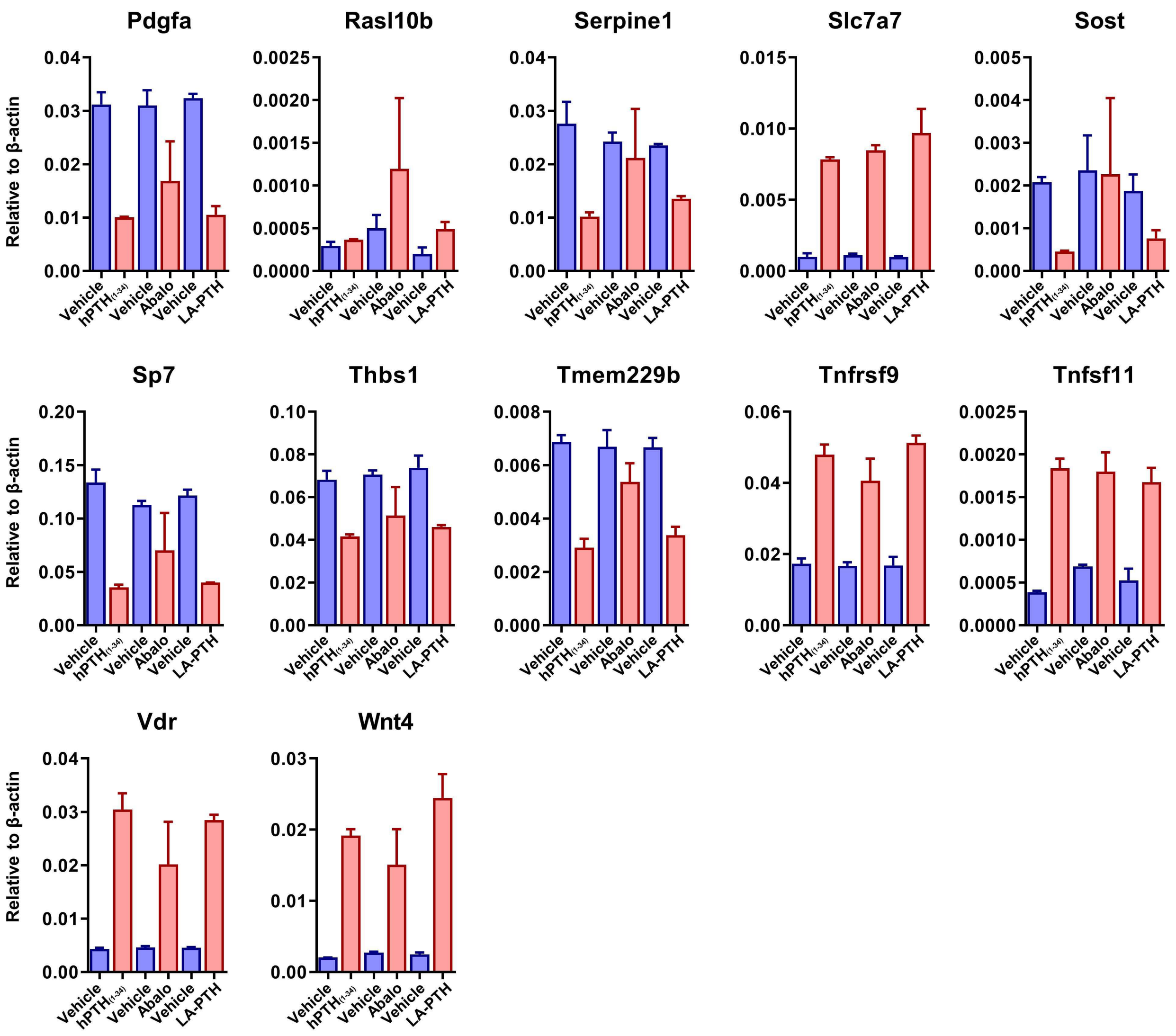

**Supplementary Figure 3:**
Ocy454 cells were treated with 20 nM PTH analogs (hPTH (1-34), ABL and LA-PTH) for 4 hours. The expression of 32 PTH/SIK target genes were assessed by Nanostring. The expression was normalized by the house keeping gene β-actin. Most gene expression changes showed no obvious differences amongst the PTH analogs tested. Data are expressed as mean ± SD of two biologic replicates.
